# Supplementary material for: Stress-shape misalignment in confluent cell layers
Source: Nat Commun. 2024 Apr 29;15:3628. doi: 10.1038/s41467-024-47702-w (PMC11059169; doi:10.1038/s41467-024-47702-w)
Supplement: Supplementary file 1 — Supplementary Information [file 41467_2024_47702_MOESM1_ESM.pdf]

Supplementary Information: Stress-shape misalignment in confluent cell layers

Mehrana R. Nejad,[1,*] Liam J. Ruske,[1] Molly McCord,[2,3] Jun Zhang,[2,4] Guanming Zhang,[5,6] Jacob Notbohm,[2,3,4,†] and Julia M. Yeomans[1,‡]

[1] *The Rudolf Peierls Centre for Theoretical Physics, Department of Physics, University of Oxford, Parks Road, Oxford OX1 3PU, United Kingdom*
[2] *Biophysics Program, University of Wisconsin–Madison*
[3] *Department of Mechanical Engineering, University of Wisconsin–Madison*
[4] *Department of Engineering Physics, University of Wisconsin–Madison*
[5] *Center for Soft Matter Research, Department of Physics, New York University, New York 10003, USA*
[6] *Simons Center for Computational Physical Chemistry, Department of Chemistry, New York University, New York 10003, USA*

Actin fibre measurements:

Using four independent MDCK samples we fixed cells for fluorescent imaging of actin fibres. Visual observations showed that it was rarely possible to visually ascertain an unambiguous direction of the stress fibres in cells with large misalignment angle θ, whereas stress fibres were much clearer in cases where they were aligned along the long axis of the cell. This is illustrated in Fig. S3.

* mehrana@g.harvard.edu
† jacob.notbohm@wisc.edu
‡ julia.yeomans@physics.ox.ac.uk

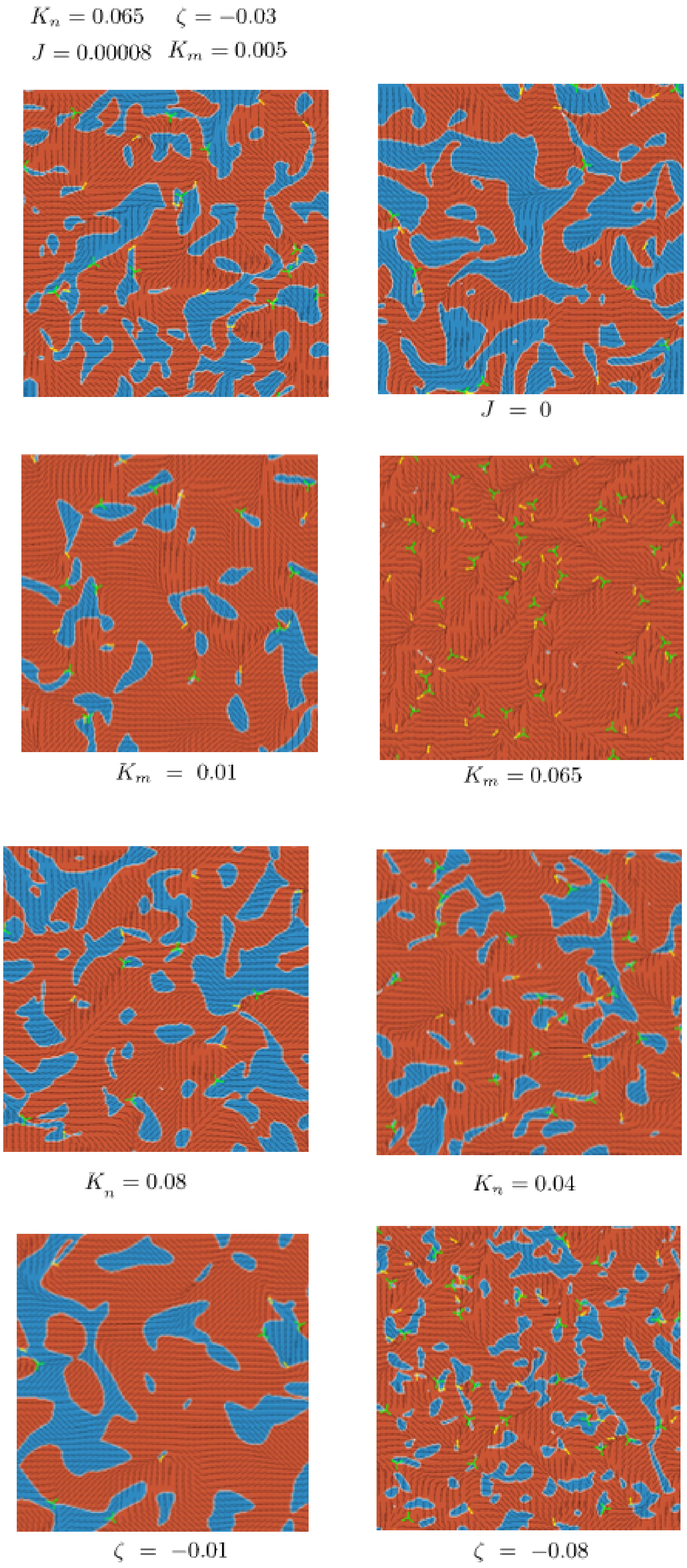

Fig. S1. The top left figure shows a snapshot of simulations for the parameter values used in the manuscript. The other sub-figures indicate that, for non-zero values of activity, extensile patches form for a wide range of values of the parameters in the simulations as long as cell shape and stress respond differently to the flows. In our model the difference comes from different elastic constants that lead to different spatial correlations ($C^n(r)$ and $C^m(r)$ in Fig. 3). When cell shape and stress have the same elastic constant ($K_m = K_n = 0.065$), the cell shape and stress respond to flows in the same manner and extensile regions do not form.

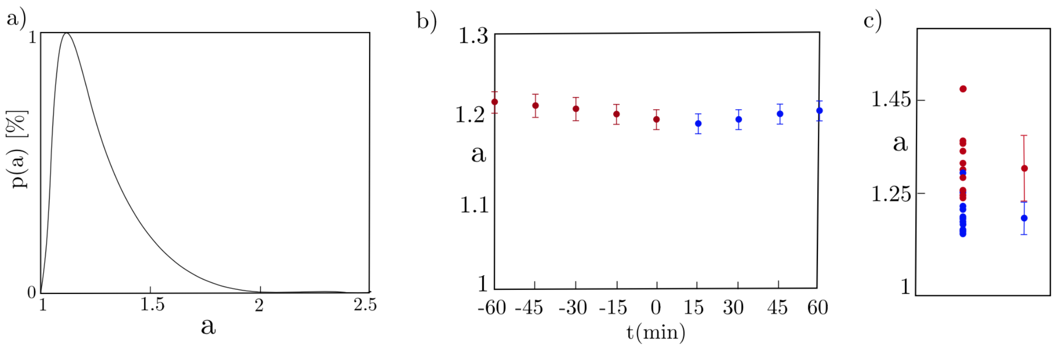

Fig. S2. a) Histogram of cell aspect ratio, a, in experiments. b) Average cell aspect ratio right before and after formation of extensile patches. The time origin is moved so that each region considered is contractile at t=0 and becomes extensile at t=15 min. Error bars show standard deviation between different experiments. The changes in the aspect ratio are small. c) Average aspect ratio of the cells in extensile (blue) and contractile (red) regions ($p = 0.0003$, rank sum test). The error bars are found using standard deviation of the average aspect ratio in different experiments. The standard deviation is equal to 0.03 (0.06) in extensile (contractile) regions. The data in (a)-(c) is averaged over 11 experiments. Source data are provided as a Source Data file in Ref. [26].

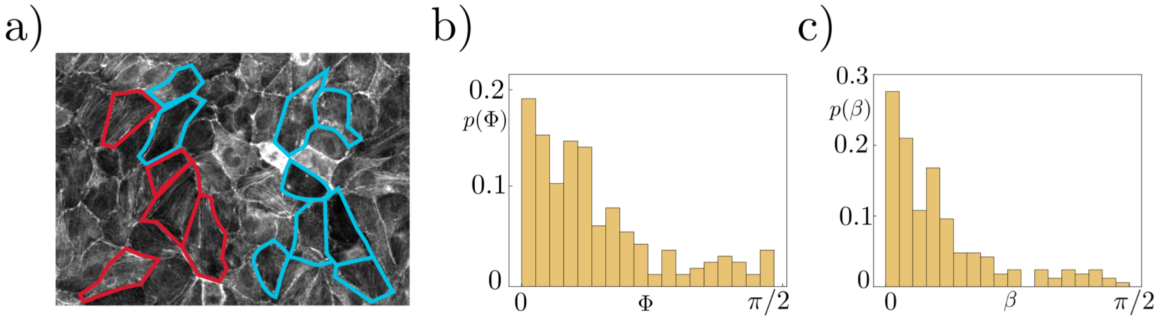

Fig. S3. (a) Enlarged snapshot of the tissue in Fig. 1 where blue (red) contours specify extensile (contractile) cells. In general, the actin is clearer in the contractile cells, where shape, the principal axis of contractile stress, and the actin tend to be aligned. (b) Probability distribution of the angle between actin and the principal axis of contractile stress Φ in cells where the actin was clear enough to enable a measurement. (c) Probability distribution of the angle between actin and the direction of cell shape elongation β in cells where the actin was clear enough to enable a measurement. Source data are provided as a Source Data file in Ref. [26].

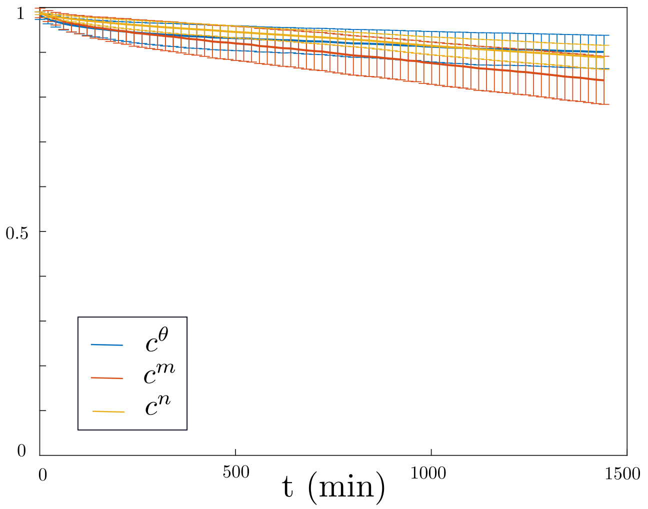

Fig. S4. Time correlations in the LP9 monolayer shows that the cell shape and stress do not change appreciably in the experiment. The data is averaged over 4 different experiments and the error bars are the standard deviation between different experiments. Source data are provided as a Source Data file in Ref. [26].

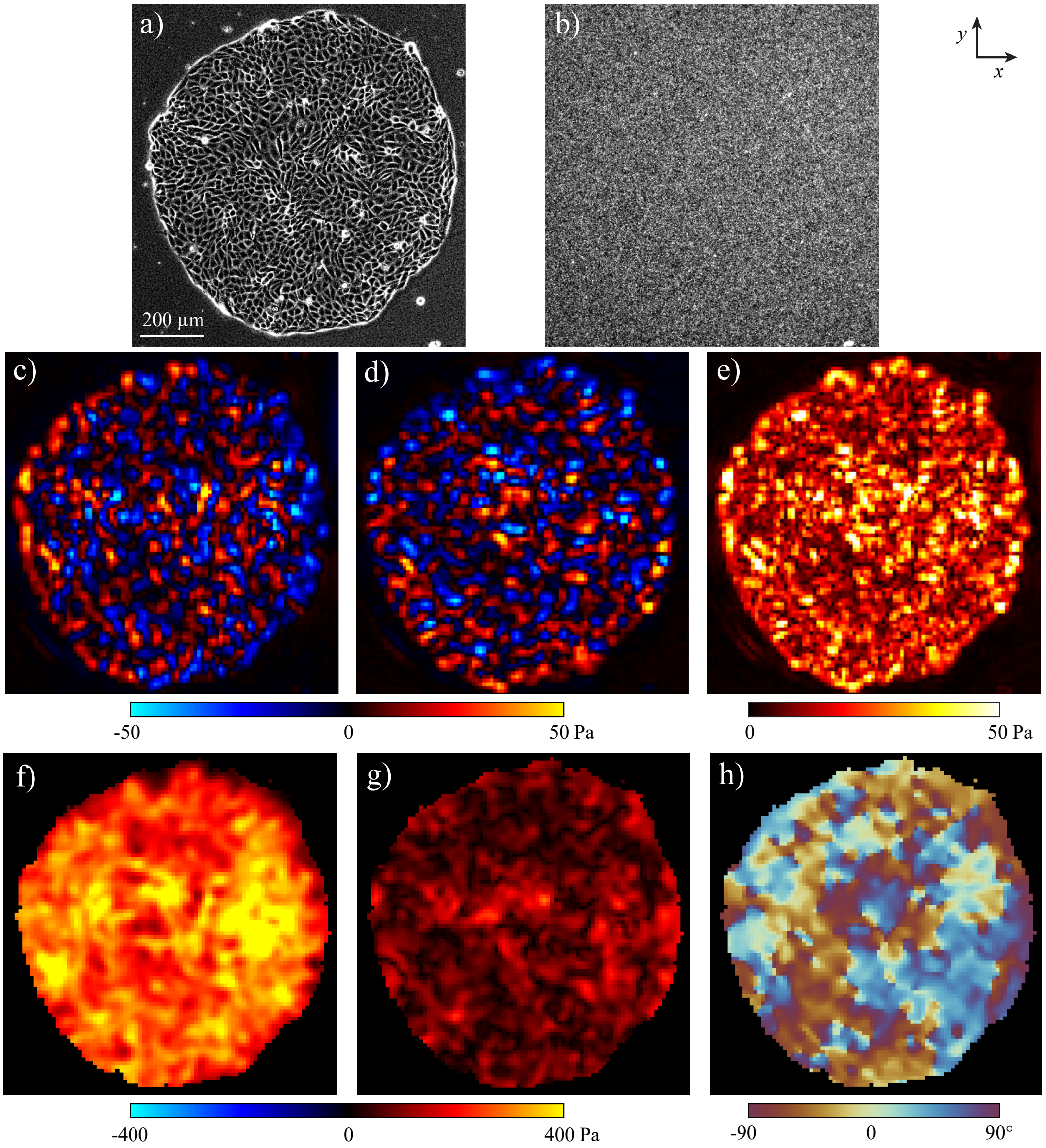

Fig. S5. Representative data from traction force microscopy and monolayer stress microscopy. (a) Representative phase contrast image of an MDCK cell island. The experiments are repeated 11 times independently with similar results. (b) Corresponding image of fluorescent particles in the substrate. (c, d) Components of traction (applied by the cells onto the substrate) in the x- (c) and y-directions. (e) Magnitude of traction. (f, g) Stresses computed from monolayer stress microscopy, namely the average normal stress, $(\sigma_1+\sigma_2)/2$ (f) and maximum shear stress $(\sigma_1-\sigma_2)/2$ (g) with σ_1 and σ_2 being the principal stresses. (h) Orientation of first principal stress. Experimental noise in the traction data is quantified by computing the root mean square (rms) of traction in locations outside the cell layer, which, for this representative image, is 1.7 Pa. The rms traction produced by the cells is 20.6 Pa, giving a signal-to-noise ratio of 12.0.
