# Supplementary material for: Stress-shape misalignment in confluent cell layers
Source: Nat Commun. 2024 Apr 29;15:3628. doi: 10.1038/s41467-024-47702-w (PMC11059169; doi:10.1038/s41467-024-47702-w)
Supplement: Supplementary file 3 — Description of Additional Supplementary Files [file 41467_2024_47702_MOESM3_ESM.pdf]

Description of Additional Supplementary Files

File Name: Supplementary Movie 1
Description: Tissue dynamics with the cell orientation field n shown as black lines on top of a colour map distinguishing contractile (red) and extensile (blue) regions. Topological defects in the cell orientation are indicated by yellow (+1/2) and green (−1/2) symbols.

File Name: Supplementary Movie 2
Description: Dynamics in the simulations, with the cell orientation field n shown as black lines on top of a colour map distinguishing contractile (red) and extensile (blue) regions. Topological defects in the cell orientation are indicated by yellow (+1/2) and green (−1/2) symbols.
